# Potentials of truffles in nutritional and medicinal applications: a review

**DOI:** 10.1186/s40694-020-00097-x

**Published:** 2020-06-17

**Authors:** Heayyean Lee, Kyungmin Nam, Zahra Zahra, Muhammad Qudrat Ullah Farooqi

**Affiliations:** 1 College of Pharmacy, Chung-Ang University, Seoul, 06974 Republic of Korea; 2 Plamica Labs, Batten Hall, 125 Western Ave, Allston, 02163 MA USA; 3 Department of Civil & Environmental Engineering, University of California, Irvine, CA 92697 USA; 4 School of Agriculture and Environment, The University of Western Australia, Perth, WA 6009 Australia

**Keywords:** Truffles, Aroma, Nutrition, Bioactive compounds, Biological activity

## Abstract

Truffles, the symbiotic hypogeous edible fungi, have been worldwide regarded as a great delicacy because of their unique flavor and high nutritional value. By identifying their bioactive components such as phenolics, terpenoids, polysaccharides, anandamide, fatty acids, and ergosterols, researchers have paid attention to their biological activities including antitumor, antioxidant, antibacterial, anti-inflammatory, and hepatoprotective activities. In addition, numerous factors have been investigating that can affect the quality and productivity of truffles to overcome their difficulty in culturing and preserving. To provide the information for their potential applications in medicine as well as in functional food, this review summarizes the relevant literature about the biochemical composition, aromatic and nutritional benefits, and biological properties of truffles. Besides, various factors affecting their productivity and quality as well as the preservation methods are also highlighted.

## Background

**Fig. 1:**
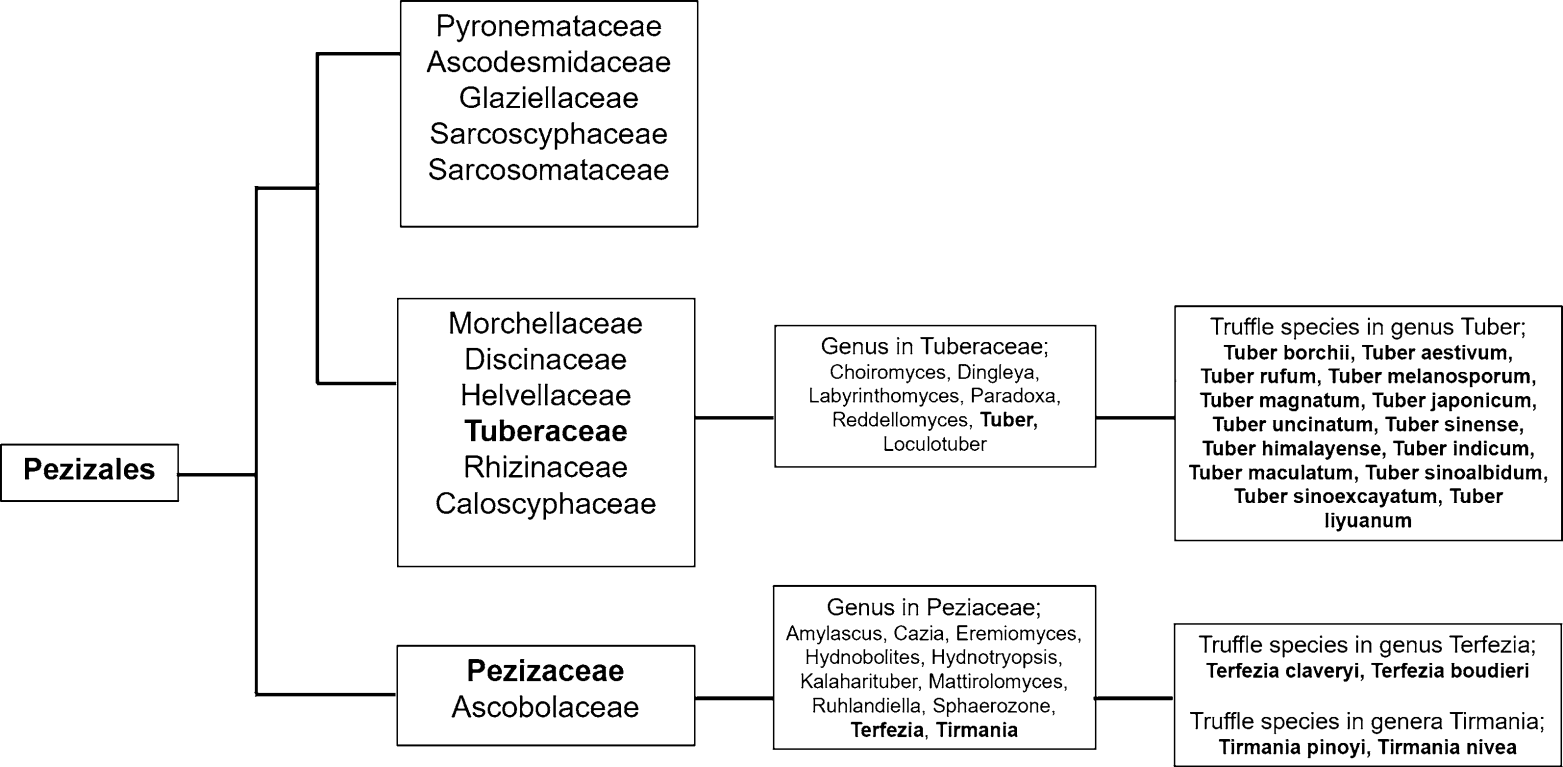
A schematic overview of the three major clades identified in Pezizales. Truffles, their genus, and family that mainly introduced in this review are shown in bold

Truffles are hypogeous ascomycetes fungi growing underground in depth between 5 and 10 cm. More than a hundred different kinds of truffle species were known worldwide, and new species are being discovered consistently. Taxonomically, edible truffles belong to family Tuberaceae and Pezizaceae, and to order Pezizales [1]. Tuberaceae is showing the diverse lineages of exclusively truffle forming fungi, and truffles belonging to this family have been widely consumed for centuries [2]. For example, the genus Tuber from the Tuberaceae family comprised of approximately more than 180 species globally [3]. Most culinary-grade truffles discovered in northern temperate forests of Europe, Australia, New Zealand, Asia, and North America generally belong to genus Tuber [4, 5]. On the other hand, desert truffles growing in the arid and semi-arid regions such as Syria, Iraq, Kuwait, Saudi Arabia, Morocco, Egypt, South Africa, and Tunisia, generally belong to the genus Terfezia and Tirmania [1, 6]. More details in the phylogeny of truffles are presented in Fig. 1.

In general, truffles tend to be firm, dense, and woody in comparison with mushrooms that is soft and fragile. White truffles include *Tuber magnatum*, *T. borchii*, *T. maculatum*, *T. oregonense*, *T. latisporum*, *T. japonicum,* and *Tirmania nivea* [7]. Among them, *T. magnatum* generally found in Italy and East Europe is the high cost truffle species [8]. In 2000 and 2001, it reached €8000 per kilogram on several Italian markets [9]. Black truffles include *Tuber melanosporum*, *T. aestivum*, *T. brumale*, *T. uncinatum*, *T. indicum*, *T. himalayense*, and *Terfezia claveryi* [7]. Truffles exhibit a large genome size. For example, *T. melanosporum* showed a genome size of 125 Mb with a high transposable element and repetitive DNA content which comprised more than 58% of the genome [10].

**Fig. 2:**
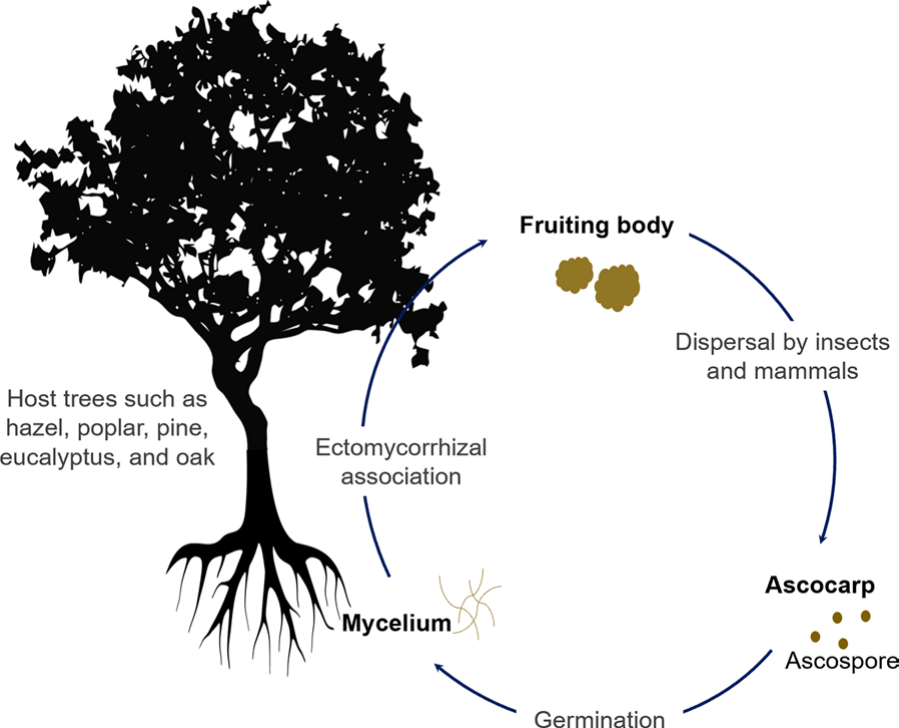
Symbiotic life cycle of truffles

Truffles are ectomycorrhizal having symbiotic root association. In a complex life cycle, the mycelia establish a symbiotic interaction with host organisms predominantly with the roots of various trees, both gymnosperms, and angiosperms, such as hazel, poplar, pine, eucalyptus, and oak [8, 11]. Because truffles produce their sexual fruiting bodies underground, the dispersal of spore is relying on insects and mammals [2]. Once ascospores are dispersed, the haploid spore germinates to haploid free-living mycelium establishing ectomycorrhizal association with the roots of host trees. Then, hyphae aggregate and form the sexual fruiting body which is an ascoma bearing asci (Fig. 2) [12–14].

The cultivation and storage of truffles are hard to control due to their dependence on several biotic and abiotic factors such as climate, humidity, soil conditions, and the surrounding flora and fauna [15]. Moreover, climate change such as increased summer temperature together with increased summer hydric stress and desertification, has led to a drop in the truffle production [16]. In France harvesting predominantly *T. melanosporum,* truffle harvest reached 2000 tons by more than 40 departments in the nineteenth century. However, in 2003–2004, the truffle production represented only ~ 10 tons mainly by Vaucluse, Dordogne, Gard, Lot, and Alpes de Haute-Provence. Nowadays, only ~ 20 tons of truffles are harvested worldwide, and the demand for truffles exceeds their supply [11, 17]. To overcome the gap between demand and supply of truffles, some commercial farming companies such as Gazzarrini Tartufi, La Maison Plantin, and La Truffe du Ventoux, have been increasing researches and developing the cultivation techniques. It has been reported that the global truffles market size will be increased by $375.3 million during 2019–2023, and Europe will account for the highest truffles market throughout this period [18].

Although truffles are regarded as a costly delicacy, they are worldwide appreciated as valuable foodstuffs due to their distinctive flavor [19, 20]. The aroma of truffles can range from mild to intense and vary from garlicky, pungent, vanilla-like, creamy, pungent, and dusty like [21]. Among hundreds of aroma active compounds in truffles, 2-methylbutanal, 3-methylbutanal, dimethyl disulphide (DMDS), dimethyl sulphide (DMS) are the most common natural aroma compounds. However, truffle derived products have a short shelf-life and are only available during the truffle season, the food industry has been developing the synthetic flavors. The exact composition of the synthetic aroma to imitate truffle flavor is unknown, even if, for example, the truffle-flavored oils contain more than 60 volatiles, of which 2,4-dithiapentane is the most common [22]. The mixture of DMS and 2-methylbutanal has been also used for a long time by the food industry to reproduce *T. melanosporum* aroma [15].

Apart from the aromatic feature, their biochemical composition encompasses multiple nutritional and medicinal benefits. Truffles are rich in various types of essential nutritional products including carbohydrates, proteins, fats, minerals, lipids, and amino acids [7]. In addition, they are rich in phenolics, terpenoids, polysaccharides, and phytosterols which are related to their antitumor, antioxidant, antibacterial, hepatoprotective, anti-inflammatory, and immunomodulatory properties [11]. In recent decades, researchers have paid great attention to the bioactive compounds derived from diverse truffle species and their potential in nutritional and medicinal applications [7, 23]. This review summarizes and updates the current status of knowledge on the chemical composition of truffles, their biological activities as well as nutritional and medicinal potential, and various factors contribute to their productivity and quality.

### Aroma profiles of truffles

The unique flavor of truffles is one of the main reasons to get worldwide attraction as a food product. Previous studies have focused on screening and identifying the volatile organic compounds (VOCs), namely the aroma active compounds, and characterized more than 200 VOCs in various truffle species. The major compounds responsible for the aroma in diverse truffle species were well documented in 2011 reviews [7, 15]. Our review aimed to provide an update on research conducted since 2011 about the analysis of active aroma components as well as the analytical techniques to identify them (Table 1).

**Table 1:** Aroma profiles of truffles

Species	Origin	Analytical methods	Observations	References
*T. nivea, P. lefebvrei, T. boudieri*	Negev desert	HS-SPME–GC–MS	Hexanal and 1-octen-3-ol were key volatiles in *T. boudieri* and *T. nivea* fruiting bodies. *P. lefebveri* with weak aroma exhibits low levels of both volatiles. *T. nivea* contains dimethyl disulfide, methional, benzaldehyde and benzenacetaldehyde, 3-methylbutanal and 2-methylbutanal. Total volatile levels in *T. nivea* are about twofold higher than in *T. boudieri*. *T. boudieri* has around tenfold higher volatile levels than in *P. lefebveri*	[25]
*T. formosanum*	n.g.	SPME–GC–MS	In *T. formosanum* mycelium, a total of 23 VOCs was identified, then quantified. Consequently, dimethyl sulfide, isopropyl alcohol, 2-butanone, ethanol, and 1,3-pentadiene were revealed as the key aroma contributors in *T. formosanum*	[26]
*T. melanosporum*	Truel, Spain	HS-SPME–GC–MS	The effect of freezing on the aroma of *T. melanosporum* ascocarps was evaluated. The frozen samples were richer in diacetyl, 1-octen-3-one, 1-octen-3-ol, 2-methylisoborneol, and dimethyltrisulphide. 1-octen-3-one was the only aroma contributor which significantly increased after the freezing process and was suggested as a potential marker for the freezing	[27]
*T. magnatum, T. melanosporum, T. aestivum*	Italy	HS-SPME–GC–MS	Aroma active compounds in 20 raw truffles, truffle-flavored oils, and truffle sauces were profiled. 1-octen-3-ol was detected in high amounts in raw *T. melanosporum*. Bis(methylthio)methane was mainly characterized both in sauces and naturally and artificially made oils flavored with *T. melanosporum*.	[28]
*T. liyuanum*	China	HS-SPME–GC–MS	A total of 57 volatiles in *T. liyuanum* were detected for the first time. Among them, 3-octanone, phenylethyl alcohol, isopentana, and methylbutana were the key contributor to the aroma. This study also suggested the optimal extraction condition for the screening of aroma active compounds: sample amount, 2 g; extraction temperature, 80 °C; extraction time, 40 min; SPME fibre of carboxen/PDMS	[29]
*T. borchii*	Italy and New Zealand	HS-SPME–GC–MS/O, GC-R, AEDA	The contribution of thiophene derivatives contributed to human-sensed aroma of *T. borchii* was investigated. A total of eleven key aroma active compounds, which showed an FD factor higher than 5, were identified. The thiophene derivatives, in particular 3-methyl-4,5-dihydrothiophene, were the main contributors to the human-sensed aroma of *T. borchii*	[32]
*T. sinensis, T. sinoalbidum, T. sinoexcavatum*	China	SPME–GC–MS/O/FPD, aroma recombination	A total of 44, 43, and 44 volatiles were detected in *T. sinensis*, *T. sinoalbidum*, *and T. sinoexcavatum*, respectively. Among them, 24 compounds were suggested as the important contributors to the overall aroma of three truffle varieties which present flora, mushroom, and sweet odor	[24]
*T. melanosporum, T. indicum*	Truel, Spain	HS-SPME–GC–MS/O	This study found the aromatic marker for distinguishing between *T. indicum* and *T. melanosporum*. 1-Octen-3-one and 1-octen-3-ol, the main aroma contributors in *T. indicum*, presented the modified frequencies (MF) of 82% and 69%, respectively, while both compounds in *T. melanosporum* exhibited the low MF (< 30%). In *T. melanosporum*, ethyl isobutyrate, ethyl 2-methylbutyrate, and isopropyl acetate contributed significantly to aroma with the high MF (> 70%)	[33]
*T. himalayense, T. indicum, T. sinense*	Yunnan and Sichuan, China	HS-SPME-GC–O	Twelve aroma active compounds, included 3-(methyl-thio)propanal, 1-octen-3-ol, 3-methylbutanal, 2-nonenal, benzeneacetaldehyde, hexanal, dimethyl sulfide, 3-methyl-1-butanol, 3-octanone, benzaldehyde, 2-phenylethanol, and dimethyl disulfide, were identified in *T. himalayense*, *T. indicum*, and *T. sinense* fruiting bodies. Among them, 3-(methylthio)propanal, 3-methylbutanal, and 1-octen-3-ol were the key contributors to the aroma in these three species	[34]
*T. magatum pico, T. uncinatum*	n.g.	HS-SPME–GC–MS/O	More than 40 odorants were evaluated as truffle volatiles for the first time. 2,3-Butanedione, 2- and 3-methylbutanal, 3-(methylthio) propanal, and bis(methylthio)-methane showed the highest FD factors in *T. magnatum pico*, while 2,3-butanedione, phenylacetic acid, and vanillin in *T. uncinatum*. 3-Methylbutanal, 3,4-dihydro-2-(H)pyrrol (1-pyrroline), and bis(methylthio)methane exhibited the highest OAV in *T. magnatum pico.* In *T. uncinatum*, 1-pyrroline and 2,3-butanedione revealed the highest OAVs	[20]
*T. japonicum, T. magnatum, T. melanosporum*	*T. japonicum* from Hyogo and Tochigi, Japan, *T. magnatum* from Italy, *T. melanosporum* from France	HS-SPME–GC–MS/O	This study compared the chemical composition of *J. Japonicum* (Japanese white-colored truffle) ascomata with *T. magnatum* and *T. melanosporum*. 1-octen-3-ol and 3-methyl-2,4-dithiapentane were highly contributed to aroma of *T. japonicum.* In addition, 3-methyl-2,4-dithiapentane was detected as a characteristic sulfur volatile in *T. japonicum* while 2,4-dithiapentane is the key odorant of *T. magnatum*	[35]
*T. melanosporum*	Sichuan, China	HS-SPME–GC-FID/O, AEDA, aroma evaluation method	The novel method exhibited their potentials as a viable alternative to the traditional method. A total of seven key volatiles in *T. melanosporum* (2,5-dimethylpyrazine, 3-butyl-2,5-dimethylpyrazine, 3-ethyl-2,5-dimethylpyrazine, 3-methyl-1-butanol, 3-(methylthio)-1-propanol, benzeneacetaldehyde, and phenylethyl alcohol) identified by the traditional method using GC–O, was also characterized by the new method along with additional two important aroma contributors, 2,3-butanediol and trimethylpyrazine	[31]
*T. magnatum Pico*	Alba and San Miniato, Italy	PTR-TOF–MS	29 volatiles were identified in this study for the first time. The VOC profiles enabled to differentiate between summer and fall/winter *T. magnatum* production as well as their geographical origin (Alba and San Miniato)	[36]
*Chinese black and white truffle*	Yunnan Province, China	DSE-SAFE coupled with GC × GC/HR-TOF–MS, electronic nose	In Chinese black truffle (BT), 14 alcohols and phenols, 13 aldehydes, 10 acids, 6 esters, 6 ketones, 5 furans and furanones, 2 hydrocarbons, and 2 sulfur-containing compounds were identified. In the white truffle (WT), 12 aldehydes, 10 acids, 9 alcohols and phenols, 5 sulfur-containing compounds, 4 furans and furanones, 3 esters, 3 ketones, and 1 hydrocarbon, were identified. More sulfur-containing compounds were detected in WT in terms of both amounts and contents	[40]
*T. magnatum*	Oils supplied from Germany, Italy, Switzerland, and UK; Fruiting bodies from Piedmont, Italy/Valjevo, Serbia/Sellye, Hungary/Piedmont, Italy	GC–MS, GC-IRMS	The differences among home-made and commercial truffle oils were evaluated. The δ13C value of 2,4-dithiapentane in most flavored oil samples was obtained by GC-IRMS and GC–MS. GC-IRMS could not distinguish between synthetic and natural flavors, while the metabolic profiling using GC–MS revealed that two sulfur containing volatiles, dimethyl sulfone and dimethyl sulfoxide, were exclusively detected in commercial oils	[22]
*T. magnatum Pico*	Italy	HS-SPME MDGC–C–IRMS	Bis(methylthio)methane was analyzed to discriminate natural and synthetic aroma. The Stable isotope ratio (δ13C) values of this volatile compound in genuine truffles attained between − 43 and − 34‰, while those from synthetic aroma exhibited more negative values	[42]

*n.g* not given

Truffles possess significant variability in their aroma profiles from species to species. In general, sulfur compounds such as dimethyl sulfide (DMS) and dimethyl disulfide (DMDS), 1-octen-3-ol, and 2-methyl-1-propanol have been identified in most truffle species including *Tuber melanosporum*, *Tuber aestivum*, and *Tuber borchii* [15]. In *Tuber magnatum* (white truffle), bis (methylthio) methane (BMTM) was reported as the most critical aroma active compound [24]. To deepen the knowledge about a complex odor of various truffle species, researchers have developed multiple methods to analyze truffle’s aroma. Traditionally, VOCs have been comprehensively profiled by solid-phase microextraction (SPME), which is generally followed by gas chromatography–mass spectrometry (GC–MS) [25–27]. Torregiani et al. analyzed 20 raw truffles, truffle sauces, and truffle-flavored oils using headspace SPME with GC–MS (HS-SPME–GC–MS). Consequently, BMTM was predominantly detected both in raw white truffles and oils flavored with white truffle but not detected or detected only in low amounts in the *T. melanosporum* (black truffle) samples; instead, 1-octen-3-ol was detected in high amounts in the raw black truffle and oil samples flavored with black truffle [28]. The HS-SPME–GC–MS technique enabled that Liu and Li profiled for the first time a total of 57 volatiles in *T. liyuanum*; a novel truffle species that have been identified in recent years. In this new truffle species, aldehydes and aromatics were the main chemical constituents, and 3-octanone, phenylethyl alcohol, isopentane, and methylbutane contributed to the significant aromaticity [29].

The GC–MS based analysis expressed limitations to determine the correlation of quantified volatiles to the olfactory stimulus as this technique could not give information about human perception. Moreover, the perceived odor presented frequently at lower concentrations than the instrumental detection limit [30]. To overcome these limitations and to identify the key aroma contributors amongst detected volatiles, the flavor dilution (FD) factor by aroma extract dilution analysis (AEDA) or/and the odor activity value (OAV) could be determined, which are based on the gas chromatography–olfactometry (GC–O) technology [31]. Splivallo and Ebeler performed the GC–O based analysis to investigate whether thiophene derivatives contributed to the human-sensed aroma in *T. borchii*. Consequently, they identified a total of 13 main aroma active compounds that showed FD factor higher than 5 and determined that thiophene derivatives; particularly in 3-methyl-4,5-dihydrothiophene, were the important contributors to the human-sensed aroma in *T. borchii* [32]. Feng et al. performed the SPME extraction combined with GC–O technology. They determined 24 compounds as key contributors to the overall aroma in three Chinese truffle varieties which present floral, mushroom, and sweet odor [24]. In addition to these studies, numerous researchers performed GC–O based analysis together with SPME–GC–MS to find the critical aroma contributor among the comprehensively profiled truffle volatiles [19, 32–34].

The olfactometry based analysis considering the entire aroma mixture has been regarded as the most useful method for estimating the contribution of key aroma-active compounds. Although it represents the standardized method for determining the aroma concentration, it does not determine the single compounds and their contribution to the aroma, and the large number of dilution of AEDA is a time-consuming process [30]. Liu et al. combined the aroma evaluation method with principal component analysis (PCA) to overcome the limitations of olfactometry based methods for fermentation samples; because the fermentation condition significantly affects aroma attributes, the changing condition one-at-a-time and the repeating AEDA and OAV are laborious. The olfactometry-based method required a total of 5278 dilution samples from 26 fermentation conditions to determine the FD, while the aroma evaluation method scored 504 fermentation samples from various fermentation conditions to identify potential aroma compounds, among them the key volatiles was determined by PCA. Consequently, seven key volatiles identified by GC–O based analysis were also characterized by the new approach along with an additional two significant aroma contributors, 2,3-butanediol, and trimethylpyrazine. The results showed the potential of an aroma evaluation method combined with PCA to study key aroma compounds of fermentation samples [31]. Vita et al. employed time-of-flight (TOF–MS) based Proton Transfer Reaction-Mass Spectrometer (PTR-MS) technology which improved the GC–MS based methods and provided a fast, accurate, and direct measurement of volatiles. This innovative technique enabled the comparison of the volatiles in *T. magnatum pico* fruiting bodies from Tuscany region with those from the Piedmont region and to distinguish the biological phases of the fruiting bodies [36]. Meanwhile, direct solvent extraction-solvent-assisted flavor evaporation (DSE-SAFE) technique has been applied in many aspects of aroma active compounds such as in fruit and wine because DSE can extract most of the volatiles as well as non-volatiles from samples. DSE, in combination with SAFE, enables the careful and fast isolation of volatiles from solvent extracts [37–39]. Zhang et al. employed DSE-SAFE coupled with comprehensive two-dimensional gas chromatography (GC × GC) high-resolution-TOF/MS to distinguish the aroma profiles of Chinese white and black and truffle. They revealed for the first time that the Chinese white truffle exhibited more sulfur-containing volatiles (2.894 µg/g in total) than the Chinese black truffle (0.040 µg/g in total) in terms of both amounts and contents based on fresh weight of samples used in extraction [40].

In recent times, researchers have increasingly recognized the authenticity and traceability of flavor compounds in truffles. The authenticity and traceability can be determined by GC coupled with combustion-isotope ratio mass spectrometry (GC–C–IRMS), which exploits ^13^C/^12^C ratio abundance of the main aroma contributor in foods [41, 42]. Wernig et al. used GC-IRMS and GC–MS to discriminate the home-made and commercial truffle oils. The GC-IRMS could not differentiate between natural and synthetic flavors, while the metabolic profiling using GC–MS revealed that two sulfur-containing volatiles, dimethyl sulfone, and dimethyl sulfoxide were exclusively detected in commercial oils [22]. To discriminate the natural aroma from the synthetic one, Sciarrone et al. evaluated bis-(methylthio)methane known as one of the key aroma contributors in *T. magnatum pico*, using the improved multidimensional GC–C–IRMS with simultaneous quadrupole MS, by minimizing the effect of column bleeding on stable isotope ratio (δ^13^C) measurement. Consequently, the δ^13^C values of bis-(methylthio) methane from genuine truffle samples attained between − 42.6 and − 33.9‰, while those from synthetic aroma exhibited more negative values [42]. More details about the recent aroma related studies are listed in Table 1 (cited on page # 35).

### The nutritional profile of truffles

Apart from the unique aroma, there are various nutritionally valuable compounds present in truffles. Although the compositional details of truffles vary from species to species and area to area, carbohydrates and proteins are generally the most abundant nutrients in truffles [1]. Besides, minerals, fibers, amino acids, fatty acids, and fats account for a significant portion of truffle composition [7, 43]. For example, a desert truffle genera *Terfezia* possessed 46–48 g/100 g of dry weight (DW) of carbohydrates, 32–35 g/100 g DW of protein, 14–15 g/100 g DW of ash, and 2.8–3.2 g/100 g DW of fat, while another desert truffle genera *Tirmania* exhibited lower protein (8–29 g/100 g DW) and ash content (5.1–5.3 g/100 g DW) but higher carbohydrate (58–83 g/100 g DW) and fat content (4–7 g/100 g DW) [44–47]. According to Yan et al., the dry matters of three Chinese truffle species, *T. latisporum*, *T. subglobosum*, and *T. pseudohimalayense*, were composed of 74–79 g/100 g carbohydrates, 11–15 g/100 g protein, 8.1–8.8 g/100 g ash, 2.2–2.5 g/100 g fat, 24–50 g/100 g total sugars, 96–265 mg/100 g monounsaturated fatty acid, 249–368 mg/100 g polyunsaturated fatty acid, and 70–121 mg/100 g of saturated fatty acid [48].

Various truffle species including *T. aestivum*, *T. borchii*, *T. magnatum*, *T. melanosporum,* desert truffles, and Chinese truffles exhibited a significant amount of unsaturated fatty acid (UFA) such as oleic acid and linoleic acid, accounting for more than 60% of their total fatty acid content [48, 49]. For example, the fatty acid in the fruiting bodies of *T. pinoyi* was mainly composed of oleic and linoleic acid, which possessed 32.29% and 29.72%, respectively [45]. Yan et al. demonstrated that linoleic and oleic acid is the most abundant fatty acid in *T. latisporum, T. subglobosum, and T. pseudohimalayense*. The dried powder of these three Chinese truffles contains 246.81–365.24 mg/100 g linoleic acid and 94.04–250.97 mg/100 g oleic acid, respectively [48]. Linoleic acid is an essential fatty acid and acts as a precursor of 1-octen-3-ol, which is one of the vital aroma active compounds in most truffle species [15, 50]. Oleic acid exhibited potentials in reducing cholesterol levels, preventing cardiovascular, and inhibiting cancer progression [51, 52]. In terms of their high UFA content, truffles might be considered as a potential nutritional and medicinal food.

Minerals are the essential dietary constituents that are required for numerous metabolic reactions, rigid bone formation, the transmission of nerve impulses, and regulation of water and salt balance [53]. Truffles provide a significant amount and variety of minerals. In particular, the major minerals such as potassium, magnesium, calcium, and phosphorus were abundant in European and desert truffles [44, 46, 54, 55]. Truffles are also known as a rich source of amino acids, especially the sulfur-containing amino acids (cysteine and methionine), while they generally remain limited in other plant-derived foods [56].

Extracellular enzymes play a role in nutrient acquisition and symbiotic fungus-host interaction helping truffles to penetrate and colonize into roots of the host plants [57]. *T. maculatum* produced seven extracellular enzymes, including amylase, catalase, cellulase, laccase, lipase, peroxidase, and xylanase; however, *T. aestivum* produced amylase, catalase, and peroxidase [58, 59].

Truffles can be considered as a source of protein but not appropriate for human’s daily diet because of their high price [7]. However, it is not suitable to regard them only as a costly source of protein because truffles possess great capability in both the diversity and amount of nutritionally valuable compounds such as fiber and minerals. Moreover, Bouatia et al. demonstrated that *T. pinoyi* either exhibited no or very low levels of antinutrient compounds including cyanide, oxalate, mercury, arsenic, and cadmium [47]. From this perspective, truffles can be consumed as a potential nutritional source with safety.

### Bioactive compounds in truffles

Truffles are rich in bioactive compounds such as ascorbic acid, ergosterol, phenolics, flavonoids, terpenoids, phytosterol, and polysaccharides. In particular, truffles contain abundant flavonoids that are appreciated as an important secondary metabolite of natural products due to their diverse biological properties involving antioxidative, anti-inflammatory, anti-mutagenic, and anticancer properties, while edible mushrooms cannot synthesize, therefore, do not contain these valuable metabolites [60, 61]. From this perspective, researchers have paid attention to the biological activities of truffles and their application in medicinal domains. For example, chemical analysis of three Chinese truffle species revealed that the methanol extracts of them contained abundant antioxidant sources, including 4.59–4.63 g/100 g ascorbic acid, 144–271 mg of β-carotene/100 g carotenoid, 450–735 mg gallic acid equivalents (GAE)/100 g phenolics, and 611–1355 mg of rutin/100 g flavonoid, which could be used to prevent diseases related to oxidative damage [48]. Furthermore, the desert truffle *T. nivea* exhibited relatively higher total phenolic content (1.39 g GAE/100 g fresh mass (FM)) as compared to other phenolic-rich fruits such as cherries (44–88 mg GAE/100 g FM), strawberries (54–94 mg GAE/100 g FM), or onions (142–428 mg GAE/100 g FM). In particular, their methanolic extract comprised a high number of flavonoids (74.52 mg GAE/g extract) and tannins (19.78 mg catechin equivalent (CE)/g extract). These abundant bioactive compounds in truffle extract were correlated to the 2,2-diphenyl-1-picryhydrazyl (DPPH) radical-scavenging and lipid peroxidation inhibitory properties together with the antimicrobial properties against seven species of bacteria [62–65]. Ergosterol, the biological precursor of vitamin D_2_, is also one of the abundant bioactive compounds in diverse truffle species [66]. Villares et al. reported that *T. melanosporum, T. aestivum,* and *T. indicum* presented total ergosterol amount of 1.90 mg/g DM, 1.86 mg/g DM, and 1.37 mg/g DM, respectively [67]. This bioactive compound and its derivatives have exhibited various health-promoting activities including antioxidant, anti-inflammatory, and antihyperlipidemic activities [68–70].

### Biological activities of truffles

#### Antitumor activity

Recently, many research studies have been conducted to find out the anticancer properties of truffles. According to Beara et al., methanol extracts from *T. aestivum* and *T. magnatum* possessed significant in vitro cytotoxic effects against different cancer cell lines (HeLa, MCF-7, and HT-29), besides the prominent activity of the water extract towards breast adenocarcinoma (MCF-7) [71]. In another study conducted by Khadri et al., silver nanoparticles were synthesized from aqueous extract of *T. claveryi*, which is particularly rich in amino acids and flavonoids. The synthesized nanoparticles exhibited significant in vitro cytotoxic effects (IC_50_ = 10 mg/mL) against MCF-7, which was evaluated by the colorimetric sulforhodamine B assay [72]. Using MTT (3-[4, 5-dimethylthiazol-2-yl]-2, 5 diphenyl tetrazolium bromide) assay, Dahham et al. determined the significant in vitro cytotoxic effects of hexane and ethyl acetate extract obtained from *T. claveryi* on different cancer cell lines, including the human brain carcinoma cell line (U-87 MG) with IC_50_ values of 50.3 µg/mL DW of extract. Furthermore, this study showed the anti-angiogenic efficacy of hexane extract using rat aorta ring assay, the hexane extract inducing the reduction of mitochondrial membrane potential as well as the condensation of nuclei in U-87 MG cells. Their anticancer properties would be correlated to their bioactive compounds including stigmasterol, β-sitosterol, squalene, and lupeol [73]. Phytosterols played a role in the inhibition of cancer cell growth mechanism, angiogenesis, and stimulation of cancer cell apoptosis [74]. Stigmasterol, one of the major phytosterols, downregulated lipid peroxidation and increased the levels of glutathione, superoxide dismutase, and catalase in the liver, and consequently decreased tumor volume, viable cell count, and increased mean survival time of Ehrlich ascites carcinoma tumor-bearing mice [75]. Squalene involved in natural triterpene potentially inhibited cancer cell proliferation without disturbing the normal biochemical pathways [76].

Truffles comprised significant amounts of polysaccharides possessing anticancer activity. Most polysaccharides in fungi are β-glucan polymers having the main chain which contains β-(1 → 3) linkage with some β-(1 → 6) branches [77]. Polysaccharides and their derivates play important roles in various biological processes, including pathogenesis prevention, signal recognition, and cell–cell communication [78]. It has been reported from previous studies that these macromolecules have oncogenesis- and metastasis-preventing abilities, including immunopotentiation activity against tumors. The polysaccharides in truffles could stimulate lymphocyte and macrophage division and synthesis of cytokines such as interferons, interleukins, and immunoglobulins directed against cancer antigens [79–81]. According to Attia et al., polysaccharides extract from *T. claveryi* possessed anticancer effect on Ehrlich’s ascites carcinoma cells, arresting G2 phase and reducing the percentage of G0/G1 phase in the cell cycle [82]. Zhao et al. revealed that 52 polysaccharides isolated from *T. aestivum*, *T. indicum*, *T. melanosporum*, and *T. sinense* fermentation systems as well as from their fruiting bodies showed antitumor activities against A549, HCT-116, HepG2, HL-60, and SK-BR-3 cells lines. This study found that the fraction from the fermentation system, which contained β-d-glucan having triple helix conformation (high-chain stiffness), showed significantly higher cancer cell inhibition rate than those from fruiting bodies. Moreover, the heteropolysaccharides with having lower molecular weight exhibited higher antitumor activity than those with higher molecular weight [83].

The oleic acid content in truffles also contributed to the anticancer activities. Oleic acid could suppress the overexpression of HER2, an oncogene correlated to the invasion and metastasis of cancer cells. Moreover, they could increase the intracellular ROS production and caspase 3 activity and consequently induce the cancer cell apoptosis [84, 85].

Pacioni et al. found that the fruiting bodies of *T. melanosporum* contained the major endocannabinoid metabolic enzymes such as *N*-acylphosphatidylethanolamine-specific phospholipase D (NAPE-PLD), fatty acid amide hydrolase (FAAH), diacylglycerol lipase (DAGL) and monoacylglycerol lipase (MAGL), and anandamide which is a fatty acid neurotransmitter derived from arachidonic acid and showed anticancer activity [86]. According to Picardi et al. [87], anandamide inhibited the angiogenesis of highly invasive and metastatic breast cancer cells. Patsos et al. [88] revealed that PG-EAs, the cyclooxygenase 2 (COX-2) dependent metabolites of anandamide, induced apoptosis of colorectal cancer cells, while anandamide stimulated non-apoptotic cell death in COX-2 overexpressed colorectal cancer cells. In EGFR-overexpressed prostate cancer cells, anandamide decreased EGFR levels by interacting with cannabinoid CB1 receptor, and consequently, inhibited the cancer cell proliferation [89].

#### Antioxidant and anti-inflammatory activities

Truffles comprise of secondary antioxidant metabolites such as phenolics, flavonoids, tocopherol, carotenoids, and phytosterols. As a free radical scavenger, phenolics can be used as an efficient antioxidant due to their reducing abilities as hydrogen- or electron-donating agents [90]. According to Beara et al. [71], water and methanolic extracts of *T. aestivum* and *T. magnatum* possessed moderate antioxidant properties related to their phenolic contents of 12–18 and 13–19 mg GAE/g DW, respectively. According to Villares et al., the methanolic extract of *T. aestivum*, *T. indicum*, and *T. melanosporum* comprised phenolics and ergosteryl ester of 1.88 mg/g DW and 0.36 mg/g DW, 1.52 mg/g DW and 0.09 mg/g DW, and 1.20 mg/g DW and 1.10 mg/g DW, respectively. Consequently, the highest phenolics and ergosteryl ester levels of *T. aestivum* were closely correlated to its greatest antioxidant power amongst these three species [67].

Desert truffle species also comprised of diverse antioxidant secondary metabolites. *T. nivea* showed much higher total phenolic content than that of *T. pinoyi*, and possessed greater antioxidant potential than *T. pinoyi* [19, 46]. According to Hamza and Jdir, ascorbic acid content (10.63 mg/100 g), and the combined content of carotenoids (1.17 mg/100 g), and anthocyanins (29.1 mg/100 g) in *T. nivea* were responsible to their antioxidant potential [46]. Doğan and Aydın revealed that the high scavenging effect of *T. boudieri* would correlate with catechin, the predominant phenolics present in this truffle [91].

Apart from desert truffles, Chinese truffle species also possessed high antioxidant properties. Chen et al. [92] evaluated the great DPPH radical scavenging ability of purified water-soluble polysaccharides from *T. huidongense*. Yan et al. demonstrated that *T. latisporum*, *T. subglobosum*, and *T. pseudohimalayense* revealed antioxidant activities, in particular, *T. latisporum* and *T. pseudohimalayense*, which were more abundant in phenolic compounds, possessed much stronger antioxidant potential than *T. subglobosum* [48].

The antioxidant compounds including phenolics, terpenoids, and polysaccharides also possess anti-inflammatory properties [77, 93]. Oxidative stress is often correlated to inflammatory disorders, particularly diabetes mellitus (DM). It is due to the activity of hyperglycemia, the typical indicator of DM, causes over-production of free radical [94]. Zhang et al. evaluated the effects of aqueous extract from *T. melanosporum* on the streptozotocin induced hyperglycemic rat. Consequently, the rat treated with truffle extract showed the reduced levels of glucose like the rat treated with the standard antidiabetic drug, glibenclamide. The hypoglycemic effect of truffle extract was significantly correlated with Nrf2 and NF-kB pathways, and the regulation of enzymatic and non-enzymatic antioxidant including superoxide dismutase, catalase, vitamin E, and vitamin C [95]. In addition to hypoglycemic effect, Janakat and Nassar revealed the hepatoprotective effect of aqueous extract from *T. claveryi* on liver damage caused by oxidative stress [96]. Moreover, Beara et al. [71] demonstrated that *T. magnatum* extract inhibited the COX-1 and 12-LOX pathway products, 12(S)-hydroxy-(5Z,8E,10E)-heptadecatrienoic acid (12-HHT), thromboxane B_2_ (TXB_2_), and 12(S)-hydroxy-(5Z,8Z,10E,14Z)-eicosatetraenoic acid (12-HETE), which overexpressed in various inflammatory diseases.

#### Antimicrobial activity

The antimicrobial activity of desert truffles has been well reported for a few decades. Janakat et al. [97] demonstrated that the aqueous extract obtained from *T. claveryi* inhibited the growth of *Staphylococcus aureus* by 66.4%. According to Owaid et al., silver nanoparticles synthesized by *Tirmania* sp. revealed the antibacterial activity towards gram-negative and gram-positive bacteria, predominantly the infectious bacteria of the eyes, *Pseudomonas aeruginosa* [98]. In another study conducted by Casarica et al., the aqueous extract from *T. claveryi* possessed the antimicrobial properties against *Escherichia coli*, *Staphylococcus epidermidis*, and *Staphylococcus aureus*, and consequently, their usage was suggested to treat eye infections caused by those three bacterial species [99]. Another desert truffle, *T. nivea*, from Tunisia arid zone was also exhibited by the remarkable inhibitory activity against three species of gram-positive and four species of gram-negative bacteria including *Salmonella typhimurium*, *Escherichia coli*, *Pseudomonas aeruginosa*, *Enterococcus faecalis*, *Staphylococcus aureus*, *Staphylococcus epidermis*, and *Bacillus subtilis* [46]. Dib-Bellahouel and Fortas found that the growth of *Bacillus subtilis* and *Staphylococcus aureus* was inhibited by ethyl acetate extract of *T. pinoyi* which contained pyrazines and its derivatives as the antimicrobial active compounds [100]. Stojković et al. [45] revealed that the aqueous and methanolic extract from *T. pinoyi* efficiently inhibited the growth of *Staphylococcus aureus* in chicken soup kept in the refrigerator and at room temperature. Table 2 summarizes more details about studies investigating the antimicrobial activity of truffles.

**Table 2:** Antimicrobial activity of truffles

Truffle species	Sample	Tested microorganisms	References
*T. claveryi*	5% aqueous extract	*Staphylococcus aureus*	[97]
*Tirmania* sp.	5 mg/well silver nanoparticle	*Pseudomonas aeruginosa*	[98]
*T. claveryi*	2–3:1 (v/w) aqueous extract followed by aqueous dilution (extract/water 1:5 v/v)	*Escherichia coli, Staphylococcus epidermidis, Staphylococcus aureus*	[99]
*T. nivea*	0.3–2.1 mg/mL chloroform and methanol extract; 2–5 mg/mL petroleum ether extract; 5–8 mg/mL macerate and hot water extract	*Salmonella typhimurium, Escherichia coli, Pseudomonas aeruginosa, Enterococcus faecalis, Staphylococcus aureus, Staphylococcus epidermis, Bacillus subtilis*	[46]
*T. pinoyi*	Ethyl acetate extract	*Bacillus subtilis, Staphylococcus aureus*	[100]
*T. pinoyi*	Aqueous extract containing 0.02% Tween 80 and 5% DMSO; 30% methanolic extract	*Staphylococcus aureus*	[45]

*DMSO* dimethyl sulfoxide

#### Aphrodisiac activity

Traditionally, truffles have been used as an effective sexual enhancer due to their constituent of androstenol as a steroidal pheromone [101, 102]. While truffle hunting, animals might recognize the odor of this chemical marker. Androstenol was also found in the underarm perspiration of men and urine of women and increased sexual attractiveness higher [11, 102]. The alcoholic extract of *T. boudieri* increased the levels of luteinizing hormone and testosterone significantly in rats and consequently possessed the aphrodisiac activity owing to its androgen enhancing properties [103]. However, Al-Damegh demonstrated that the androgenic property of truffles was more related to psychological effect because the flavonoids in truffles were present in the form of glycosides, which act as antagonists for male sex hormones [102].

#### Other potential applications

The bioactive compounds in truffles potentiate the use of truffles in other medicinal usages such as anti-depressants, cholesterol reducer, and immunostimulant. For example, the blood cholesterol-reducing effect of linoleic acid has been widely reported [104, 105]. In the meanwhile, truffles comprise abundant amino acids involving l-tyrosine which is a precursor of neurotransmitters catecholamines deeply involved in neural circuits [44, 106, 107]. Pattanayak et al. studied the cytotoxic effect of water-soluble heteroglycan obtained from *T. rufum* on human blood lymphocytes. Consequently, they discovered the potential of this polysaccharide as an immunostimulant, in terms of its ameliorative activities by maintaining the redox balance [108].

### Various factors affecting productivity and quality of truffles

Like most edible fungi, the growth pattern, nutritional quality, and biological activities of truffles could be changed by various factors such as climate, soil, or the presence of contaminating microorganisms [8]. For example, dehydrative low-temperature stress induces accumulation of dehydrins in truffles proposed to play a protective role [109] and upregulation of stress-responsive genes including heat shock proteins and cell wall remodeling genes in *T. melanosporum* [110]. High temperatures and hydric stress in summer, together with decreased summer precipitation, could also lead to the genetic or morphological change and reduction of subsequent winter harvest [111]. Leonardi et al. evaluated the effect of heat stress on eleven *T. borchii* strains from different geographical and ecological provenance. Ten from eleven tested strains showed the significant growth reduction accompanying by increased hyphal septation at 34 °C, while the best performances of mycelial growth and biomass production were at 22 °C. In terms of global climate change, a suitable selection of strain which is tolerant in temperature change could be one of the successful strategies for truffle cultivation [112].

Edaphic factors such as pH, moisture, and nutrient also influence the richness and composition of truffles [113]. Hall et al. [114] demonstrated that a soil pH of 8.0–8.5 was an appropriate condition for the *T. melanosporum* cultivation. Ge et al. revealed that soil pH is strongly correlated to the levels of minerals in truffles such as K^+^, Ca^2+^, and Mg^2+^, and host plants generally produced more *T. lyonii* fruiting bodies at soil pH of 6.6–7.3 [115].

Meanwhile, truffle productivity and quality could be improved by regulating preharvest factors. As truffles are hypogeous fungi, they interact with soil micro-fauna and microorganisms, which can enhance the truffle formation and their antagonistic, competitive, or synergistic activities [8]. Frey-Klett et al. demonstrated that co-inoculating, adding a small amount of helper bacterium to the ectomycorrhizal fungus, could lead to much easier and faster-growing than ectomycorrhizal fungi. Thus, this method can be one of the efficient methods for optimizing the mycorrhization techniques as well as minimizing the inoculation cost [116]. Tang’s research team has developed and improved the submerged fermentation process for enhancing not only truffle biomass but also the production of polysaccharides, VOCs, and sterols in truffles [117]. For example, they demonstrated that the addition of Mg^2+^ and K^+^ could enhance the production of extracellular polysaccharides during the submerged fermentation of *T. sinense*. According to Vahdatzadeh and Splivallo, genetic features deeply influenced the truffle aroma formation, and the human nose could distinguish the flavor among the strains. From this regard, strain selection could be effectively used for improving the truffle flavor [118].

Truffles are highly perishable and very sensitive to pathogen contamination and water loss [119]. During storage processes such as frozen or sterilization, the aroma quality and texture of truffles had been changed. Therefore, preservation methods play a critical role in the maintenance or improving their quality. Saltarelli et al. [55] recommended a storage temperature at 4 °C to preserve the biochemical and microbiological characteristics of fresh *T. aestivum*, *T. magnatum*, and *T. melanosporum*. Al-Ruqaie demonstrated that freezing after blanching in 4% boiling NaCl solution is the most effective preservation method for maintaining the quality of *T. claveryi* and *T. nivea* [120]. Rivera et al. recommended lyophilization as an adequate method to better preserve the aroma of *T. melanosporum*, while Palacios et al. suggested that rehydration after lyophilization recovered the aromatic profile changed through lyophilization [121, 122]. According to Tirillini et al. [123], the addition of 10% linoleic acid (w/w) significantly reduced total bacterial load and enhance the nutritional value of truffle sauce.

Irradiation is one of the ways affecting microbial populations, shelf life, chemical composition, and sensory characteristics of truffles. Adamo et al. [124] demonstrated that the irradiated truffles showed a microbial load of five orders of magnitude less than the non-irradiated. Reale et al. [125] revealed that irradiation at 1.5 kilo gray (kGy) was the most effective way for extending the shelf life of truffles as well as minimizing the alteration of sensorial features of fresh truffles. According to Culleré et al. [126], the ionizing radiation at 1.5 and 2.5 kGy increased the levels of practically all the primary odorant of *T. melanosporum* and *T. aestivum*, and therefore, these type of irradiation were considered as an effective way for maintaining the truffle aroma. Tejedor-Calvo et al. recommended that electron beam or gamma irradiation (both at 1.5 kGy and 2.5 kGy) followed by storage up to 21 days were the efficient methods to enhance truffles quality and shelf life. In the study, the irradiation exhibited no influence on the total carbohydrates, β-glucans, and overall sterols levels. Although this method significantly reduced protein and chitin levels, storage after the irradiation maintained the levels of all determined compounds unchanged except for the phenolics and ergosterol levels that increased after 21 days storage [127].

Storing fresh truffles under high carbon dioxide conditions is also associated with their quality. Mencarelli et al. [128] revealed that CO_2_ treatment on *T. aestivum* at 5 °C could keep well their fragrance and robust defense system. According to Hajjar et al., storing *T. uncinatum* in high CO_2_ and low O_2_ atmospheres played a decisive role in maintaining their antioxidative properties by inhibiting polyphenol metabolism, anaerobic pathways, and polyamine biosynthesis. The antioxidant enzymes in truffles were active when they are kept in the soil with lower O_2_ conditions; therefore, removing truffles from lower to higher O_2_ conditions could weaken their antioxidant defense system [119].

## Conclusions

The unique flavor of truffles has made them the high demanded foodstuffs; however, they are not enough to be regarded only as high-priced foodstuffs because of their nutritional and medicinal potentials as well as no known side effects. Diverse truffle species are rich in nutrients such as carbohydrates, protein, fiber, minerals, fatty acids, and amino acids. Apart from their high value in the nutritional domain, their potential in therapeutic agents is ample as well. Recent studies have identified diverse bioactive compounds such as phenolics, flavonoids, and polysaccharides in truffles and revealed their potentials as anticancer, antioxidant, antimicrobial, anti-inflammatory, antidiabetic agents. Nevertheless, most studies have focused on in vitro studies and limited researches on the application of truffles in practical therapy has been investigated. Future studies need to optimize the extraction process to obtain a high yield of the bioactive compounds. Moreover, it is needed to focus on their bioassay against different diseases and pathogens to incorporate these biological values effectively into the truffles derived natural products as well as potential medicines. In this regard, more innovative methods are also expected to overcome the difficulty in preserving truffles to enhance their shelf life whilst maintaining their bioactive compounds and causing no harmful effects. These attempts would enable the practical usage of truffles in nutritional and medicinal fields.

## Data Availability

Not applicable.
